# Taurine Protects Doxorubicin-Induced Hepatotoxicity via Its Membrane-Stabilizing Effect in Rats

**DOI:** 10.3390/life13102031

**Published:** 2023-10-09

**Authors:** Esra Gedikli, Veysel Özgür Barış, Nilgün Yersal, Adnan Berk Dinçsoy, Sevda Fatma Müftüoğlu, Ayşen Erdem

**Affiliations:** 1 Department of Physiology, Hacettepe University Faculty of Medicine, 06230 Ankara, Turkey; e.gedikli_61@hotmail.com (E.G.); brkdincsoy@gmail.com (A.B.D.); 2 Department of Cardiology, Dr Ersin Arslan Research and Education Hospital, 27010 Gaziantep, Turkey; veyselozgurbaris@gmail.com; 3 Department of Histology & Embryology, Gaziosmanpaşa University Faculty of Medicine, 60030 Tokat, Turkey; nilgun.yersal@gop.edu.tr; 4 Department of Histology & Embryology, Hacettepe University Faculty of Medicine, 06230 Ankara, Turkey; smuftuog@hacettepe.edu.tr

**Keywords:** doxorubicin, hepatotoxicity, taurine

## Abstract

Background: Doxorubicin (dox) is a chemotherapeutic agent widely used against various tumors. However, the clinical use of this agent is limited due to various organ toxicities. Taurine is an intracellular free β-amino acid with antioxidant properties. The present study investigated the protective mechanism of taurine on dox-induced hepatotoxicity. Methods: In total, 31 male Sprague-Dawley rats were used in the study. The control group received intraperitoneal (i.p.) 0.9% NaCl alone for 14 days; the taurine (Tau) group received i.p. taurine 150 mg/kg body weight/day for 14 days; the dox group received dox on days 12, 13, and 14 at a cumulative dose of 25 mg/kg body weight/3 days; and the tau+dox group received taurine and dox together at the same dose and through the same route. On day 15, biochemical evaluations were performed on blood samples taken from the left ventricle followed by histological examinations on liver samples. Results: Dox was found to increase liver function enzymes and tissue protein carbonyl levels, causing congestion and tissue damage, thereby leading to dysfunction. Tau was found to histologically preserve the liver morphology without showing any corrective effect on oxidative stress parameters. These findings suggest that the membrane-stabilizing effect of taurine may be more effective than its radical scavenging activity in preventing dox-induced toxicity. Conclusion: Taurine can prevent doxorubicin-induced hepatotoxicity through non-antioxidant pathways.

## 1. Introduction

Doxorubicin (Dox) is an antineoplastic agent from the group of anthracyclines used in the treatment of cancer [1]. Although widely used in clinical practice, this agent has toxic side effects that limit its use. The heart is a primary target in dox-induced toxicity and evidence suggests that this toxicity may also affect other organs such as the liver, kidney, and brain [2]. The chemical structure of dox causes free radical formation and oxidative stress induction as well as an imbalance between reactive oxygen species (ROS) and antioxidants [3]. These effects lead to lipid peroxidation and protein oxidation, eventually resulting in tissue damage [4]. Studies have shown that dox causes hepatotoxicity by disrupting the hepatic antioxidant system [5]. During treatment with dox, the liver takes up, accumulates, and metabolizes doxorubicin at high concentrations. Therefore, the liver is one of the organs most affected by the administration of dox [6]. Dox administration may cause histopathologic changes such as hepatocyte degeneration and pleomorphism, bile duct proliferation, parenchymal necrosis, central vein occlusion, thrombosis, and portal inflammation, resulting in elevated serum alanine aminotransferase (ALT) and aspartate aminotransferase (AST) levels [7]. Therefore, there is a need for protective agents that can prevent or alleviate the hepatotoxicity caused by dox administration. 

Taurine (2-aminoethanesulfonic acid) (tau) is an amino acid found in high concentrations in the brain, heart, liver, and kidney of mammals and plays a role in several processes including the antioxidant process, osmoregulation, cell plasma membrane stabilization, detoxification, and bile acid conjugation [8]. It protects against free radical-induced damage in biological systems such as the heart, liver, and kidney [9]. Dox is a chemotherapeutic agent exerting its effects by causing oxidant injury and cytotoxicity. The rationale of our study was to elucidate the oxidant–antioxidant and cytotoxic–cytoprotective struggle between doxorubicin and taurine in a rat model of doxorubicin-induced hepatotoxicity. The aim of the present study was to histologically demonstrate the protective effect of taurine on dox-induced hepatotoxicity based on oxidative stress parameters, liver function tests, and light and electron microscopic examinations. 

## 2. Materials and Methods

In our study, we used 31 male Sprague-Dawley rats weighing 300–400 g which were provided by the Experimental Animals Unit of Hacettepe University, Ankara, Turkey. The rats in all groups were fed with standard chow and an unlimited amount of water. During the acclimatization period and experiments, all rats were kept in a 12-h light/12-h dark environment in a ventilation-controlled room with a constant temperature and relative humidity at the Experimental Animals Unit. The experiments were conducted with the permission of Hacettepe University Faculty of Medicine Experimental Animals Local Ethics Committee number 2015/34-06 in accordance with the Declaration of Helsinki and the Guide for the Use and Care of Laboratory Animals of the American Public Health Organization. 

Animals were randomly assigned into four groups and weighed. Control (C) group (*n* = 7): The rats in this group received 0.9% NaCl solution intraperitoneally (i.p.) for 14 days. Tau group (*n* = 8): The rats in this group received i.p. taurine (Sigma, Chemical Co., St. Louis, MO, USA) 150 mg/kg body weight/day for 14 days [10]. Dox group (*n* = 8): Rats in this group received i.p. doxorubicin (Adrimisin 50 mg, Deva, Turkey) on days 12, 13, and 14 of the experiment at a cumulative dose of 25 mg/kg (a total of 25 mg/kg body weight, i.e., day 12: 7.5 mg, day 13: 7.5 mg, and day 14: 10 mg) [11]. Tau+dox group (*n* = 8): Rats in this group received i.p. taurine 150 mg/kg body weight/day for 14 days in addition to doxorubicin injections at the same dose, using the same protocol as the dox group on days 12, 13, and 14. 

Tau and dox were dissolved in 0.9% NaCl solution. Volume adjustments were made for each drug to 1 mL per kg and the total volume of injections was adjusted to not exceed 2 mL/kg/day. All procedures were performed between 09:30 and 10:00 in the morning in order to minimize any circadian changes.

After reweighing the rats between 09:30 and 10:00 am on day 15, anesthesia was induced with ketamine (90 mg/kg, i.p.) and xylazine (10 mg/kg, i.p.). Blood was collected from the left ventricle using a cardiac puncture to assess liver function. Immediately thereafter, the liver tissue was excised and weighed. A portion of the liver tissue was fixed in formaldehyde for light microscopy and in glutaraldehyde for electron microscopy (EM). The remaining liver tissue was placed in liquid nitrogen and then stored at −80 °C until biochemical analysis. After anesthesia, all surgical procedures were completed within 2 min. However, because one of the animals in the dox group died during anesthesia, blood and tissue samples from this animal were excluded from the analyses. 

After the blood samples were centrifuged, the serum portion was separated and stored at −20 °C until analysis of serum alanine aminotransferase (ALT), alkaline phosphatase (ALP), and aspartate aminotransferase (AST) (Beckman Coulter AU680 Autoanalyzer Application Kit, Beckman Coulter, Inc., Brea, CA, USA).

The liver tissue samples were homogenized in Tris buffer (pH: 7.4). Analyses of the protein carbonyl, catalase, total oxidant, and antioxidant status were performed on these homogenates. Protein analysis was performed according to the Lowry method. Standard solutions were prepared for protein analysis and protein levels in the homogenate were determined according to the corresponding graph.

### 2.1. Protein Carbonyl Assay

Protein carbonyl (PC) levels, which represent a marker of protein oxidation, were measured to assess oxidative stress. Protein oxidation was evaluated by measuring the amount of 2,4-dinitrophenylhydrazone formed after the reaction of 2,4-dinitrophenylhydrazine with carbonyl groups (25). After homogenization, the samples were mixed with 20% trichloroacetic acid (TCA) and then centrifuged. The supernatant was removed, 10 nM 2,4-dinitrophenylhydrazine was added to the remaining pellets, and the tubes were incubated at room temperature. At the end of the incubation, 20% TCA was added to terminate the reaction. This step was followed by centrifugation and then the supernatant was discarded and the pellet was separated. The pellet was washed 3 times with an ethanol–ethyl acetate mixture. This was followed by dissolution with 100 mM NaOH at 37 °C for 15 min. At the end of this incubation, the samples were centrifuged and the supernatant was read at 360 nm against a blank. Results were reported as nmol/mg protein.

### 2.2. Catalase Activity 

Catalase (CAT) activity was monitored spectrophotometrically according to Aebi’s method [12]. The principle of the assay is based on the determination of the rate for the H_2_O_2_ decomposition at 240 nm at room temperature. One unit of enzyme activity (katal) was defined as the amount of enzyme that decomposes 1 mmole of H_2_O_2_ per minute in 50 mM phosphate buffer at pH 7.0. Results are expressed per protein in each sample (k/g protein).

### 2.3. Total Antioxidant Status (TAS)

TAS levels were measured using commercially available kits (RL0017, Rel Assay, Gaziantep, Turkey). In this method, the characteristic color of a more stable ABTS (2,2′-azino-bis (3-ethylbenzothiazoline-6-sulfonic acid)) radical cation is bleached off by antioxidants. The test has high sensitivity with an error margin of less than 3%. Results are expressed as mmol Trolox equivalent/L.

### 2.4. Total Oxidant Status (TOS)

TOS levels were measured by commercially available kits (RL0024, Rel Assay, Gaziantep, Turkey). This method is based on the oxidants present in the sample oxidizing the ferrous ion-o-dianisidine complex to ferric ion. The oxidation reaction was increased by a plethora of glycerol molecules available in the reaction medium. The ferric ion formed a colored complex with xylenol orange in an acidic medium. The color intensity was related to the total amount of oxidant molecules present in the sample. This could be measured spectrophotometrically. Hydrogen peroxide was used to calibrate the assay. Results are expressed in micromolar hydrogen peroxide equivalents per liter (μmol H_2_O_2_ equivalent/L).

### 2.5. Oxidative Stress Index (OSI)

The oxidative stress index (OSI) is one of the criteria used to evaluate oxidative stress. The oxidative stress index is calculated as the ratio of total oxidant status (TOS) (μmol H_2_O_2_ equivalent/L) to total antioxidant status (TAS) (mmol Trolox equivalent/L).

### 2.6. Histopathological Evaluations

Liver tissue samples were fixed in 10% buffered neutral formalin. The samples were transferred to the processor of the Leica TP1020 (Leica Biosystems, Nussloch, Germany). After dehydration and clearing, samples were embedded in paraffin using a Leica Eg1150H (Leica Biosystems, Nussloch, Germany) embedding station and cut into 5 μm sections. These sections were stained with hematoxylin–eosin (H&E) and examined under a Leica DM 6000 (Leica Biosystems, Wetzlar, Germany). Three different areas were evaluated at three different magnifications (×40). The criteria for liver damage were as follows: vascular congestion, sinusoidal narrowing, granular degeneration in hepatocytes, vacuolation of hepatocytes, mononuclear cell infiltration, pyknotic nuclei, and heterochromatic nuclei in hepatocytes. Histopathologic findings were graded as (−) no damage, (+) minimal damage, (++) moderate damage, and (+++) severe damage. 

Liver tissues were quickly cut into small pieces of 1 mm^3^ and fixed with 2.5% glutaraldehyde and 1% osmium tetroxide. Semithin sections of 1 μm were stained with methylen blue-azur II for light microscopy and then 70 nm sections were cut and stained with uranyl acetate-lead citrate for electron microscopic imaging (JEM-1400 (JEOL, Tokyo, Japan)).

### 2.7. Statistical Analysis

Statistical analyses were performed with IBM^®^ SPSS Statistics v20.0 (IBM Corp., Armonk, NY, USA, 2011). The difference between the groups was evaluated with the Kruskal–Wallis/post-hoc Dunn’s test at the *p* < 0.05 level. The data are represented as a median with the interquartile range (IQR).

## 3. Results

### 3.1. Body Weight and Liver Weight of Animals

All rats were weighed before and after the injections. Baseline body weight, final body weight, and liver weight per 100 g body weight were measured in all animals (Table 1). No difference was found between the baseline body weights of the rats. The difference between the initial and final body weight measurements of the rats was evaluated as the change in body weight in grams. The change in body weight was significantly different between the dox group and the tau group (*p* = 0.008) while there was no significant difference between the other groups. Liver weight was increased in both dox (*p* < 0.01) and tau (*p* < 0.05) groups. No difference in liver weight was observed between the tau+dox group and the control group.

### 3.2. ALP, ALT, and AST Activity

While dox administration decreased serum ALP levels compared to the control group (*p* < 0.05) it was found to cause a significant increase in ALT and AST levels (*p* < 0.01). Tau administration had no effect on these enzyme levels (Table 2).

### 3.3. PC Level and CAT Activity in Liver Tissue

Tissue PC levels were higher in the dox and tau+dox groups compared to the control group (*p* < 0.05). Taurine treatment did not prevent this dox-induced increase. The CAT activity was increased in the Taurine group; however, the difference was not significant compared to the control group. In addition, the CAT activity was also higher in the taurine group than in the tau+dox group (*p* < 0.01) (Table 2) (File S1).

### 3.4. Determination of Total Antioxidant Status (TAS), Total Oxidant Status (TOS), and Oxidative Stress Index (OSI) 

In the dox group, TOS and OSI levels were higher while TAS levels were lower compared to the control group (*p* < 0.01). Tau administration did not show a significant effect on these levels (Table 2). 

### 3.5. Histopathological Examination

**Figure 1:**
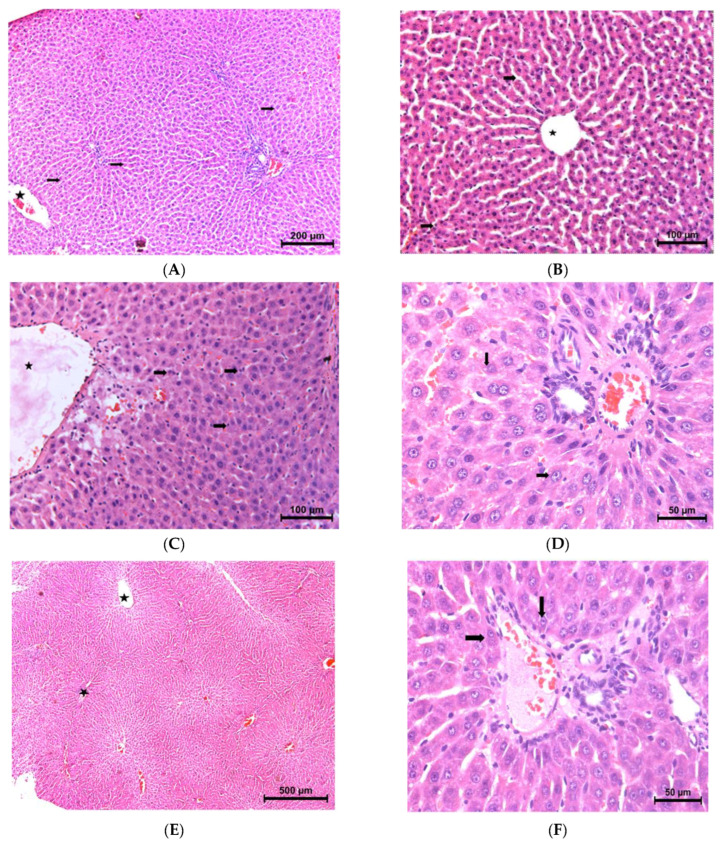
Light microscopic image of liver tissue [H&E]. For all groups; asterisk: central vein. (**A**) Control group, arrow: hepatocyte cords [×10]. (**B**) Taurine group, arrow: sinusoidal areas [×20]. (**C**) Doxorubicin group, arrow: infiltrative cells [×20]. (**D**) Doxorubicin group, arrow: vacuoles [×40]. (**E**); Doxorubicin+taurine group [×5], (**F**). Doxorubicin+taurine group arrow: limiting plate cells [×40].

Light Microscopic Features: In the control group, hepatocytes were observed as fairly regular cords extending from the central vein to the center of the lobule; no degenerative changes were observed in these hepatocytes. Sinusoidal spaces between hepatocyte cords were evident. Hepatocyte cords also appeared quite regular in the taurine group. Narrowing was observed in the sinusoidal areas and was more pronounced in the periportal region, suggesting that it may be a result of minimal cellular edema. Significant central vein congestion was observed in the dox group. Accordingly, there was enlargement in the sinusoidal areas near the central vein. The increase in infiltrating cells was remarkable compared to the other groups (*p* < 0.001). Degenerative changes were noted in hepatocytes and degenerative hepatocytes with pyknotic nuclei were particularly noted in limiting plate cells (*p* < 0.001). Vacuolar degeneration was observed in some hepatocytes (*p* < 0.001). In the dox+tau group, there was limited congestion in many central veins and no significant degeneration of limiting plate cells (Figure 1). In addition, cell infiltration, sinusoidal narrowing, and pyknotic and heterochromatic hepatocytes were significantly less than in the dox group (*p* < 0.001) (Table 3).

**Figure 2:**
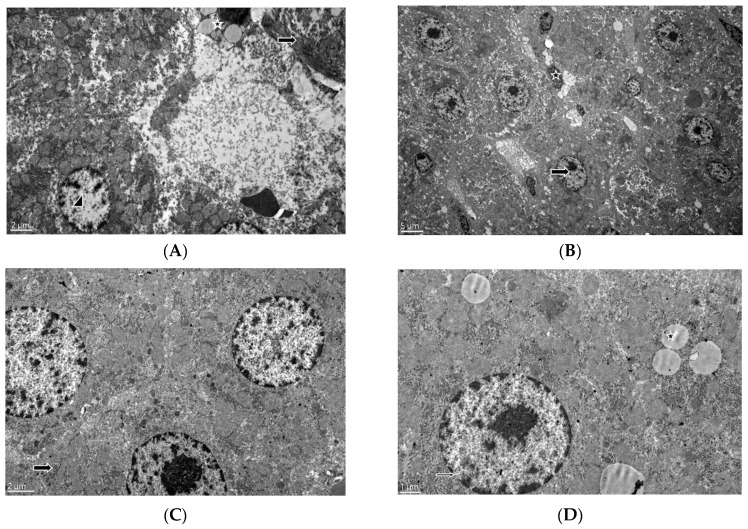

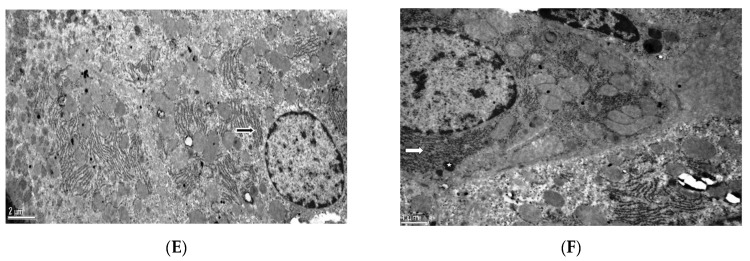
Electron microscope image of liver tissue. (**A**) Control group, arrowhead: hepatocyte nucleus, asterisk: Ito cell with fat droplets, and arrow: collagen fibers [×6000]. (**B**) Taurine group, arrow: hepatocyte nucleus and asterisk: Kupffer cell nucleus [×2500]. (**C**) Doxorubicin group, rER: tough endoplasmic reticulum and arrow: rER [×8000]. (**D**) Doxorubicin group, arrow: nuclear pore and asterisk: fat vacuoles [×12,000]. (**E**) Doxorubicin+taurine group, arrow: rER [×8000]. (**F**) Doxorubicin+taurine group, arrow: rER and asterisk: heterophagic vacuoles. [×15,000]. rER: rough endoplasmic reticulum.

Electron Microscopic Features: In the control group, there were healthy-appearing hepatocytes with patent sinusoids and normal nuclear and cytoplasmic features as well as Kupffer cells and Ito cells with fat droplets. Cellular characteristics in the tau group were similar to those in the control group. In the group treated with doxorubicin, both large numbers of fat vacuoles in hepatocytes and the enlargement of nuclear pores in hepatocytes and Kupffer cells were observed. The rough endoplasmic reticulum had a scattered appearance in the cytoplasm. On the other hand, in the dox+tau group, the rough endoplasmic reticulum formed clumps and was located close to the nucleus, as observed in the control group (Figure 2).

## 4. Discussion

This study investigated the protective effect of taurine on dox-induced hepatotoxicity and showed that taurine can prevent histological changes in dox-induced hepatotoxicity by reducing the number of infiltrating cells, central venous congestion, limiting plate cell degeneration, vacuolization, sinusoidal narrowing, and rough endoplasmic reticulum destruction. However, we observed that it achieves this protective effect without causing any significant changes in biochemical and oxidative parameters.

Doxorubicin is a chemotherapeutic agent used in the treatment of solid tumors and hematologic cancers [6]. In addition to cardiotoxic effects, it may also cause nephrotoxicity and hepatotoxicity [13]. Taurine is a sulfur-containing amino acid found in most mammalian tissues. This amino acid mediates several physiological functions such as calcium transport, osmoregulation, cell membrane stabilization, and detoxification [8]. Studies have shown that taurine has a protective effect on liver tissue [14]. Due to the fact that taurine has antioxidant activity and scavenges reactive oxygen species and thereby exerts a protective effect on liver tissue, we investigated the protective effect of taurine against dox-induced hepatotoxicity. To this end, we evaluated protein oxidation; the activity of catalase, TAS, and TOS; and histopathologic changes to evaluate morphologic changes in liver tissue. We observed that taurine had a corrective effect on ALT but not on AST levels and did not affect PC levels, one of the oxidative stress parameters. On the other hand, histological examinations showed that although taurine treatment reduced the degenerative changes induced by doxorubicin administration, it did not show sufficient efficacy in preventing the oxidative effects of doxorubicin hepatotoxicity, which is defined as liver damage associated with liver dysfunction caused by exposure to a drug or other various agents [15]. The liver performs many important functions in the body and is also responsible for the biotransformation and detoxification of drugs as well as their conversion into forms that can be easily eliminated from the body [16]. Therefore, continuous exposure to drugs is considered one of the main causes of liver damage. Doxorubicin x is among the agents that cause damage in the liver [17]. Due to its chemical structure, doxorubicin increases the formation of free radicals and also reduces the cell’s ability to detoxify reactive oxygen species [18]. 

To prevent cellular damage caused by reactive oxygen species, organisms employ enzymatic and non-enzymatic antioxidant defense systems [19]. Glutathione (GSH) is an important antioxidant produced normally in the metabolic process and protects cells against the harmful effects of oxidation products [20]. Decreased GSH levels lead to a disruption of cellular defenses and contribute to tissue damage [21]. On the other hand, taurine administration plays a role in reducing oxidative stress by stimulating endogenous antioxidants and directing cysteine to GSH synthesis [22], thereby directly increasing GSH levels [23]. However, taurine biosynthesis is reduced in liver disease [24]. For this reason, we started taurine treatment prior to doxorubicin administration to reduce the toxic effects of doxorubicin in the liver and to boost the compromised antioxidant defenses that may be a result of decreased taurine synthesis. 

Doxorubicin causes clinical signs such as weight loss, decreased activity, and acid production. Weight loss is observed in both single and long-term treatment regardless of the duration of doxorubicin administration [25]. In addition, doxorubicin administration causes physical fatigue, decreased activity, and loss of appetite in animals [25]. Taurine administration prevents weight loss and tissue weight changes [26]. In our study, we observed a decrease in the body weight of animals in the dox group. This decrease in body weight may be due to the direct toxic effects of doxorubicin on the intestinal mucosa and a decrease in appetite [26]. Moreover, we observed an increase in liver weight in dox-treated rats. In the literature, the groups that found a decrease in liver weight did not compare liver weight to body weight [27]. We used liver weight per 100 g body weight to eliminate the effect of body weight and observed an increase in liver weight, especially in the dox group [28]. This weight gain may be due to dox-induced liver congestion as observed by light microscopy. As taurine treatment reduced liver congestion and cell degeneration, liver weights in this group approached control values. Interestingly, we also observed an increase in liver weight in the group receiving taurine alone. Light microscopic findings in this group may indicate mild edema in the liver tissue caused by taurine and an increase in liver weight due to this congestion. However, when administered with doxorubicin, taurine was observed to prevent the increase in tissue weight by exerting a protective effect on the liver.

Serum transaminase levels are known to be sensitive markers of liver damage. Damage to hepatocytes alters their transport function and membrane permeability, resulting in leakage of enzymes from damaged cells. This leakage causes a decrease in hepatocyte ALT and AST levels and an increase in serum ALT and AST levels [29]. In general, elevations in serum ALT and AST levels are observed in all liver diseases. The increase is more pronounced in cases of advanced cell necrosis. In our study, we observed that ALP levels were decreased, while serum AST levels were increased approximately 2.5-fold and ALT levels were increased approximately 4-fold in the dox-treated group. Bulucu et al. also reported similar reductions in serum ALP levels in the dox group. However, in contrast to our study, they found a decrease in ALT and AST levels [13]. ALP is an enzyme found at high levels in liver, bone, placenta, and kidney tissue. An increase in ALT and AST is observed in liver damage, and an increase in ALP is most commonly observed in cholestasis, where the bile ducts are blocked and bile flow is restricted [30]. This shows that doxorubicin caused severe hepatocyte damage in our study. Similar to our results, there are many studies in the literature reporting increased serum ALT and AST levels with doxorubicin injection [7]. However, in some studies, doxorubicin is seen to cause a significant increase in serum AST levels without an increase in ALT levels [31]. 

ALT is an enzyme found in the cytoplasm of hepatocytes. High levels of this enzyme in serum provide evidence that cell death occurs due to impaired membrane permeability [32]. Because ALT levels are low in tissues other than the liver, an increase in serum ALT is considered a strong indicator of liver damage. AST is another enzyme that shows hepatocyte damage [32]. In addition to the liver, AST is found in high concentrations in the kidney, pancreas, red blood cells, heart, and skeletal muscle. Damage to any of these tissues releases AST into the bloodstream, resulting in elevated serum AST levels. The increased serum aminotransferase activity observed during doxorubicin administration results from leakage that occurs as a result of advanced damage, such as inflammatory cell infiltration and hepatocyte necrosis, triggered by dox-induced damage to hepatocyte membranes [27]. There are studies in the literature reporting that taurine decreased ALT and AST activity in liver tissue in hepatotoxicity induced by various agents [33]. In our study, although taurine brought ALT levels closer to the levels seen in the control group, it did not show a corrective effect on AST levels. However, we observed that taurine treatment significantly reduced central venous congestion, cell infiltration, limited platelet cell degeneration, the number of hepatocytes with pyknotic and heterochromatic nuclei, vacuolization, and sinusoidal narrowing and preserved rough endoplasmic reticulum morphology. Also, the space of the mall and the periportal area were not as wide as in the dox group. 

In our study, when degenerative changes were scored and converted into quantitative data, we found high scores in the dox group. This finding provides structural confirmation that doxorubicin causes toxicity in liver tissue. Dox-induced degenerative changes in hepatocytes were significantly reduced by taurine administration. 

Taurine may have exerted this effect by regulating Na^+^–K^+^ ATPase and Ca^+2^ -ATPase activities. Doxorubicin administration reduces the activity of these enzymes, resulting in membrane damage [34]. Therefore, this finding may be due to the fact that taurine provides membrane stabilization in hepatocytes, preventing ion leakage and water influx, thus protecting the cell from swelling [35]. Another possible mechanism may be the inhibition of intrinsic (mitochondrial), extrinsic receptor-mediated, and apoptotic pathways such as the endoplasmic reticulum. Doxorubicin administration causes an increase in Fas, caspase-8, and Bax levels and a decrease in Bcl-2 levels and mitochondrial membrane potential. Taurine treatment prevents these changes [34]. In addition, the increase in active calpain 1 (protein level) and caspase-12 (protein level) and the intracellular calcium concentration return to normal levels with taurine administration [34]. Electron microscopy images from this study show that taurine protects cellular structures such as the endoplasmic reticulum. Increased intracellular calcium is known to cause disruption of the structure of the endoplasmic reticulum [36]. In our study, the correction of dox-induced intracellular organelle perturbations by taurine treatment demonstrated the importance of taurine in calcium homeostasis. Although this study is the first to demonstrate the protective role of taurine in dox-induced hepatotoxicity by electron microscopy, we did not measure intracellular calcium concentrations which is a limitation of our study. 

Since oxidative stress is determined based on the rate of formation of oxidant molecules and the total potency of all antioxidant molecules, examining antioxidant molecules individually may be insufficient to demonstrate the total intracellular oxidative stress (59). Therefore, we measured TAS and TOS levels as well as the oxidative stress index in our study. These measurements showed that the TAS level was significantly lower and that the TOS level and oxidative stress index were higher in the dox group. However, these measurements in the tau+dox group were similar to those in the dox group. This finding indicates that the antioxidant capacity of taurine was insufficient against the effect of doxorubicin in our study. 

The semiquinone form of doxorubicin is a short-lived metabolite that interacts with molecular oxygen and initiates the reaction cascade that produces reactive oxygen species [1]. This semiquinone radical causes the production of hydroxyl radicals, hydrogen peroxide, and superoxide anions which lead to lipid peroxidation [37].

Reactive oxygen species attack polyunsaturated fatty acids in membranes as well as proteins and genetic material. When evaluated together, PC and antioxidant capacity represent one of the key parameters showing the extent of tissue damage in doxorubicin-induced hepatotoxicity [38]. 

It has been reported in the literature that taurine supplementation interferes with cyclosporine A-induced inhibition of CAT and increases CAT activity [39]. Furthermore, it has been reported that taurine administered orally at 100 mg/kg body weight to reduce the hepatotoxic effects of tamoxifen, a cytotoxic chemotherapeutic agent, decreased PC levels in mouse liver tissue and increased CAT enzymatic activity [40]. PC levels are one of the most commonly measured products of protein oxidation. Determination of the carbonyl level is important in the detection of oxidative protein damage [41]. In our study, we found that doxorubicin increased PC levels in rat liver tissue. However, taurine did not show a reducing effect on PC levels in liver tissue in our study. We also examined the activity of catalase, an enzymatic antioxidant, and found increased CAT activity in the group of rats that received taurine alone, although it was not significantly different from the control group. 

Taurine shows direct antioxidant activity by scavenging free oxygen radicals and also shows an indirect antioxidant effect to control the increase in membrane permeability caused by oxidative stress in the liver [42]. Insufficient antioxidant capacity may be the reason why taurine failed to prevent protein oxidation in our study. Protein damage is an indicator of an intense attack [42,43]. On the other hand, although methionine and cysteine are highly sensitive to oxidative attack, their regulation and treatment do not always result in a corrective effect on protein function [42]. This may explain the mechanism of the failure of taurine treatment to improve protein oxidation in our study. 

Dox-induced toxicity is not limited to ROS damage. It may also occur through a number of known but unresolved mechanisms, such as topoisomerase II inhibition, the induction of intrinsic, extrinsic, and endoplasmic reticulum-mediated apoptotic pathways; lipid peroxidation; and inhibition of PGC-1α. Nagai et al. reported that taurine exerts a protective effect against doxorubicin independent of its antioxidant activity, with a decrease in Fas and Bax mRNA expression levels. 

Although in our study we found that the scavenging effect of taurine was not sufficient to prevent the oxidative effect of doxorubicin, the correction of certain biochemical parameters and histopathologic findings in liver tissue suggests that taurine exerts a protective effect through antioxidant-independent pathways.

We showed the protective effect of taurine against doxorubicin-induced hepatotoxicity by measuring the total antioxidant and oxidant status and by electron microscopic examination at the organelle level, which are the strengths of this study. However, among the limitations of our study, we could not measure the inducing or inhibiting effect of taurine on cytochrome P450 and glutathione S transferase enzymes. Also, since we recruited only male rats in order to exclude the estrus-dependent variations in female rats, we could not evaluate the gender-related differences in the toxicity of doxorubicin and the effect of taurine on it. In cases of hepatotoxicity, the principal metabolic process involves the biotransformation of xenobiotics, particularly by cytochrome P450 enzymes and glutathione S transferase. This metabolic pathway exhibits variations contingent upon the gender of the subjects involved. Studies indicate that there are gender-specific variations in hepatic cytochrome P450 function in rats [44,45,46]. This situation is of great importance in explaining the inter-gender drug-related differences in disease progress and treatment success.

## 5. Conclusions

This study has histologically demonstrated the protective effects of taurine against doxorubicin-induced hepatotoxicity. The fact that this protective effect precedes the antioxidant effects of taurine suggests that taurine also acts through other pathways. Histologic changes suggest non-antioxidant pathways. According to our literature search, the present study is the first to demonstrate the protective effect of taurine against doxorubicin-induced hepatotoxicity by electron microscopy. For the general use of taurine to prevent dox-induced hepatotoxicity, our findings should be confirmed by further clinical trials.

## Figures and Tables

**Table 1:** Body Weight and Liver Weight of Animals (g).

	C (*n* = 7)	Tau (*n* = 8)	Dox (*n* = 8)	Dox+Tau (*n* = 8)
Body weight at baseline (g)	377.09 ± 23.47	333.00 ± 26.14	358.75 ± 36.82	360.00 ± 34.43
Difference in body weight (g)	−4.16 ± 12.42	8.43 ± 27.64	−30.09 ± 18.56 ^b^	−21.65 ± 18.02
Liver weight/100 g b.w. (g)	2.74 ± 0.07	3.04 ± 0.26 *	3.07 ± 0.33 ^#^	2.93 ± 0.09

*: (*p* < 0.05) vs. group C, ^#^: (*p* < 0.01) vs. group C, ^b^: (*p* < 0.01) vs. group Tau. C: Control, Tau: Taurine, Dox: Doxorubicin, n: number of animals, b.w.: body weight.

**Table 2:** Serum (s) and Liver Tissue (t) Biochemical Parameters.

	C (*n* = 7)	Tau (*n* = 8)	Dox (*n* = 7)	Dox+Tau (*n* = 8)
ALP(s) (U/L)	194.86 ± 30.59	205.88 ± 44.30	128.29 ± 23.50 *^,a^	135.75 ± 21.61 ^a^
ALT(s) (U/L)	39.43 ± 2.70	46.63 ± 4.60	161.00 ± 49.70 ^#,a^	155.38 ± 57.59 ^#^
AST(s) (U/L)	104.71 ± 16.31	146.38 ± 30.02	276.57 ± 81.54 ^#^	328.75 ± 128.93 ^#,a^
PC(t) (nmol/mg protein)	7.81 ± 6.99	14.76 ± 7.50	16.79 ± 6.49 *	16.54 ± 5.24 *
CAT(t) (k/g protein)	6.23 ± 1.91	9.49 ± 2.13	6.04 ± 1.24	5.26 ± 1.98 ^b^
TAS(t) (mmol Trolox Equivalent/L)	5.22 ± 1.34	5.61 ± 0.78	3.02 ± 4.33 ^#^	3.14 ± 0.32
TOS(t) (μmol H_2_O_2_ Equivalent/L)	12.23 ± 2.38	11.27 ± 1.39	20.58 ± 3.99 ^#^	18.5 ± 1.46
OSI (TOS/TAS)	2.52 ± 0.87	2.04 ± 0.35	6.82 ± 1.05 ^#^	6.00 ± 1.00 ^#^

*: (*p* < 0.05) vs. group C, *^#^:* (*p* < 0.01) vs. group C, ^a^: (*p* < 0.05) vs. group tau, ^b^: (*p* < 0.01) vs. group tau C: Control, Tau: Taurine, Dox: Doxorubicin, n: number of animals. ALP: Serum Alkaline Phosphatase, ALT: Alanine Aminotransferase, AST: Aspartate Aminotransferase, PC: Protein Carbonyl, CAT: Catalase, TAS: Total Antioxidant, TOS: Total Oxidant, OSI: Oxidative Stress Index.

**Table 3:** Histopathologic evaluation of liver sections.

	C (*n* = 7)	Tau (*n* = 8)	Dox (*n* = 7)	Dox+Tau (*n* = 8)
Granular degeneration of hepatocytes	0.06 ± 0.16	0.13 ± 0.35	3.13 ± 0.35 ^c^	1.3 ± 0.59 ^e^
Mononuclear cell infiltration	0.33 ± 0.43	0.63 ± 0.69	2.75 ± 0.46 ^c^	0.88 ± 0.23 ^e^
Sinusoidal narrowing	0.28 ± 0.44	0.69 ± 0.70	2.00 ± 0.53 ^c^	0.75 ± 0.46 ^e^
Vascular congestion	0.22 ± 0.44	0.50 ± 0.46	2.25 ± 0.46 ^c^	1.19 ± 0.75 ^d,c^
Vacuolation of hepatocyte	0.06 ± 0.17	0.19 ± 0.37	2.62 ± 0.52 ^c^	1.06 ± 0.68 ^e,c^
Pyknotic nuclei and heterochromatic nuclei in hepatocyte	0.22 ± 0.44	0.38 ± 0.52	2.88 ± 0.35 ^c^	1.38 ± 0.52 ^e,c^

^c^: (*p* < 0.001) vs. group C, ^d^: (*p* < 0.01) vs. group dox, ^e^: (*p* < 0.001) vs. group dox. C: Control, Tau: Taurine, Dox: Doxorubicin.

## Data Availability

All histological images (Figure 1 and Figure 2) and data results (Table 1, Table 2 and Table 3) are original data. The histological images and raw datasets analyzed during the current study are available on request from Aysen Erdem (aerdem@hacettepe.edu.tr).

## Supplementary Materials

The following supporting information can be downloaded at: https://www.mdpi.com/article/10.3390/life13102031/s1, File S1: Spectrophotometric raw data of CAT activity.
