# Supplementary material for: Taurine Protects Doxorubicin-Induced Hepatotoxicity via Its Membrane-Stabilizing Effect in Rats
Source: Life (Basel). 2023 Oct 9;13(10):2031. doi: 10.3390/life13102031 (PMC10608465; doi:10.3390/life13102031)
Supplement: Supplementary file 1 [file life-13-02031-s001.zip › life-2613455-supplementary.pdf]

Liver/CAT	0. sec	15. sec	30. sec	45. sec	60. sec
C 1	0,56	0,485	0,444	0,427	0,39
C 2	0,494	0,468	0,42	0,382	0,368
C 3	0,488	0,428	0,397	0,36	0,317
C 4	0,515	0,483	0,447	0,448	0,405
C 5	0,502	0,43	0,426	0,391	0,386
C 6	0,496	0,441	0,411	0,324	0,306
C 7	0,499	0,408	0,38	0,306	0,277
Tau 1	0,496	0,423	0,373	0,311	0,292
Tau 2	0,502	0,453	0,392	0,319	0,284
Tau 3	0,494	0,436	0,336	0,292	0,274
Tau 4	0,5	0,423	0,369	0,318	0,265
Tau 5	0,504	0,424	0,403	0,341	0,281
Tau 6	0,499	0,443	0,352	0,302	0,272
Tau 7	0,504	0,431	0,349	0,301	0,259
Tau 8	0,503	0,426	0,372	0,29	0,265
Dox 1	0,516	0,467	0,414	0,363	0,336
Dox 2	0,527	0,484	0,463	0,438	0,408
Dox 3	0,558	0,491	0,442	0,415	0,383
Dox 4	0,579	0,499	0,472	0,437	0,392
Dox 5	0,527	0,476	0,433	0,416	0,38
Dox 6	0,515	0,471	0,427	0,386	0,352
Dox 7	0,521	0,451	0,419	0,345	0,287
Tau+Dox 1	0,525	0,485	0,468	0,431	0,391
Tau+Dox 2	0,545	0,482	0,442	0,417	0,385
Tau+Dox 3	0,538	0,484	0,434	0,373	0,323
Tau+Dox 4	0,521	0,473	0,444	0,442	0,4
Tau+Dox 5	0,534	0,492	0,452	0,438	0,437
Tau+Dox 6	0,52	0,461	0,411	0,405	0,378
Tau+Dox 7	0,528	0,484	0,449	0,406	0,362
Tau+Dox 8	0,555	0,486	0,461	0,414	0,343
